# Externalizing the threat from within: A new direction for researching associations between suicide and psychotic experiences

**DOI:** 10.1017/S0954579420001728

**Published:** 2021-01-06

**Authors:** Jamie Murphy, Mark Shevlin, Louise Arseneault, Richard Bentall, Avshalom Caspi, Andrea Danese, Philip Hyland, Terrie E. Moffitt, Helen L. Fisher

**Affiliations:** 1 School of Psychology, Ulster University, Ulster, Northern Ireland; 2 King’s College London, Social, Genetic & Developmental Psychiatry Centre, Institute of Psychiatry, Psychology & Neuroscience, London, UK; 3 Department of Psychology, University of Sheffield, Sheffield, UK; 4 Department of Psychology and Neuroscience, Duke University, Durham, NC, USA; 5 Department of Psychiatry and Behavioral Sciences, Duke University Medical School, Durham, NC, USA; 6 King’s College London, Department of Child & Adolescent Psychiatry, Institute of Psychiatry, Psychology & Neuroscience, London, UK; 7 National & Specialist CAMHS Clinic for Trauma, Anxiety and Depression, South London & Maudsley NHS Foundation Trust, London, UK; 8 Department of Psychology, Maynooth University, County Kildare, Republic of Ireland; 9 ESRC Centre for Society and Mental Health, King’s College London, London, UK

**Keywords:** birth-cohort, cross-lagged panel analysis, psychosis, self-harm, self-injurious behavior

## Abstract

A recent suicidal drive hypothesis posits that psychotic experiences (PEs) may serve to externalize internally generated and self-directed threat (i.e., self-injurious/suicidal behavior [SIB]) in order to optimize survival; however, it must first be demonstrated that such internal threat can both precede and inform PEs. The current study conducted the first known bidirectional analysis of SIB and PEs to test whether SIB could be considered as a plausible antecedent for PEs. Prospective data were utilized from the Environmental Risk (E-Risk) Longitudinal Twin Study, a nationally representative birth cohort of 2232 twins, that captured SIB (any self-harm or suicidal attempt) and PEs at ages 12 and 18 years. Cross-lagged panel models demonstrated that the association between SIB at age 12 and PEs at age 18 was as strong as the association between PEs at age 12 and SIB at age 18. Indeed, the best representation of the data was a model where these paths were constrained to be equal (*OR* = 2.48, 95% CI = 1.63–3.79). Clinical interview case notes for those who reported both SIB and PEs at age 18, revealed that PEs were explicitly characterized by SIB/threat/death-related content for 39% of cases. These findings justify further investigation of the suicidal drive hypothesis.

## Introduction

Associations between psychosis and self-injurious/suicidal behavior (SIB) have been repeatedly evidenced in the psychiatric research literature and continue to be a primary focus for many researchers. From 2018 to date there have been at least nine systematic reviews/meta-analyses, exploring the co-occurrence of these phenomena (Gournellis et al., 2018; Grigoriou, Upthegrove, & Bortolotti, 2018; Hettige, Bani-Fatemi, Sakinofsky, & De Luca, 2018; Hielscher, DeVylder, Saha, Connell, & Scott, 2018; Huang, Fox, Ribeiro, & Franklin, 2018; Large et al., 2018; Oakley et al., 2018; Woodrow et al., 2018; Yates et al., 2019).^1^ Major studies in the area have included large prospective cohort data analyses (Fisher et al., 2013; Kelleher et al., 2013), analyses of clinically high-risk and ultra-high-risk psychosis sample data (DeVylder, Lukens, Link, & Lieberman, 2015; Hutton, Bowe, Parker, & Ford, 2011), and analyses of general population epidemiological data (Koyanagi, Stickley, & Haro, 2015; Taylor, Hutton, & Wood, 2015). Furthermore, all have considered SIB risk and occurrence in the context of psychotic experiences (PEs) (i.e., all have attempted to explain or interpret risk for and occurrence of SIB based on the presence, severity, duration, or context of PEs). However, it seems that the association between PEs and SIB has only ever been investigated in a unidirectional framework; where SIB has always been an outcome of, but never an antecedent to, PEs.

A recent hypothesis has offered an alternative perspective and has suggested that PEs, for some, may be consequential to SIB. Given (a) the significant co-occurrence of both phenomena and the early presence of SIB among those in receipt of care for first episode psychosis, (b) the overlooked “internal threat” status of SIB, and (c) the commonly reported and recorded threat-laden content and phenomenology of PEs, proponents of a new suicidal drive hypothesis (Murphy et al., 2018) have suggested that PEs (particularly threat-informed positive PEs) might meaningfully reflect an individual’s psychology in the context of internal threat exposure such as SIB. Revisiting and reconsidering Eugene Bleuler’s (1911) proclamation that the “suicidal drive” is the “the most serious of schizophrenic symptoms” and recognizing the substantial extant research literature evidencing the diversity and complexity of defensive psychological reactions to external sources of threat, the suicidal drive hypothesis suggests that internal threat, too, may evoke unique psychological reactions that have the potential to optimize an individual’s defense and survival.

According to this hypothesis, impending harm or death, for those who pose a threat to their own safety/survival, may be delayed or prevented via some psychological process of threat externalization (i.e., PE). By attributing internal threat to sources other than, and external to themselves (which becomes manifest as, e.g., paranoia about personal safety or impending danger; persecutory delusions about the malevolent intentionality of others to cause harm, injury or death; and/or auditory verbal hallucinations that command engagement in suicidal or self-injurious behavior) those experiencing SIB may secure psychological “distance” and protection from their own thoughts, beliefs and/or behaviors that have become an imminent mortality risk. While such anomalous, threatening experiences are likely to be highly distressing, their perceived source/location as threat(s) from without, rather than from within, may also stimulate emotions and beliefs, and activate defensive behaviors, that normally and commonly promote survival in contexts where external threats are real and present. Psychosis, therefore, may essentially create the necessary context (e.g., presence of threatening agent/voice; suggestion of conspiratorial group/plot; incrimination via television/radio broadcasts) and induce the necessary psychological/emotional states (e.g., fear, anxiety, distress, panic, hypervigilance etc.) and behaviors (e.g., alarm, help seeking) needed for survival. Psychological departure, therefore, from a reality, where threat to life is inescapable, and the adoption of an alternate, where threat can be resisted/evaded, may bolster defense and sustain life in the midst of the most severe forms of threat to life (i.e., SIB).

Justification for this line of enquiry, however, first necessitates preliminary evidence that can demonstrate (a) that SIB can precede psychosis; (b) that PEs are likely to arise among those engaged in SIB; (c) that the strength of the association between PEs and SIB is likely to vary according to both SIB recency and severity; and, most importantly, (d) that PEs among those engaging in SIB are likely to be informed and characterized by SIB/threat/death-related content. The first empirical tests of the suicidal drive hypothesis, summarized briefly below, initiated this line of investigation.

### Suicidal drive hypothesis: preliminary findings

Using prospective data from a Danish population cohort, Murphy et al. (2018) first sought to test the temporal occurrence of clinically recorded SIB and psychosis. Data from a complete cohort of all individuals born in Denmark in 1984 (*N* = 54,458; 49% female) were sourced. Using the Danish Psychiatric Nationwide Case Register and the National Patient Register, annual hospital admissions data for both SIB (hospitalization for self-harm or suicidal behavior) and psychosis (annually recorded status denoting presence of schizophrenia, schizotypal or delusional disorders) were targeted specifically. In total, 406 (0.7%) individuals from the cohort had received a psychotic disorder diagnosis in their lifetime and 1034 (1.8%) had a hospital/psychiatric record denoting SIB. Of these, 82 (0.1%) individuals had a record of both psychotic disorder diagnosis and SIB treatment. Among those with dual SIB–psychosis status, 31 (37.8%) received hospital treatment for SIB and were diagnosed with a psychotic disorder in the same year; 34 (41.4%) were hospitalized for SIB in the year(s) prior to psychotic disorder diagnosis; and 17 (20.7%) were diagnosed with a psychotic disorder in the year(s) prior to SIB hospitalization. Proportionally, however, the analysis showed that SIB either preceded or co-occurred with psychosis in 79% of dual-status cases.

Second, using cross-sectional data from two UK epidemiological surveys, Murphy and colleagues tested whether PEs were more commonly reported by those who experienced SIB (SIB in this analysis = suicidal ideation and behavior) and whether the strength of the association between PEs and SIB varied according to (a) SIB recency (lifetime/last 12 months) and (b) SIB severity (suicidal thought/attempt). These analyses indicated that individuals who thought about suicide in their lifetime were up to eight times more likely to experience specific PEs while those who both thought about and attempted suicide in their lifetime and in the year of assessment were up to 48 times more likely to experience PEs compared to SIB-free members of the population. Overall, the probability of PEs increased in relation to SIB recency and severity.

While these findings offered tentative preliminary evidence of SIB → psychosis directionality, they were also limited in a number of important ways. While the Danish data were prospectively oriented and population based, they were at the same time restricted to hospital admission records only and as such did not offer an opportunity to capture either phenomenon at a subclinical level. Conversely, while the UK epidemiological data captured the subclinical expression of both phenomena, the cross-sectional nature of the data precluded the temporal ordering of SIB and PEs in a way that was necessary to more robustly test the proposed hypotheses. Neither set of analyses therefore afforded an ideal opportunity to satisfactorily test the temporal associations between these phenomena or to explore the phenomenology of psychosis symptoms for those who were engaged in or had a history of SIB.

The current study sought to address these limitations and, in so doing, more rigorously test some of the foundational assumptions underpinning the suicidal drive hypothesis. Using cohort data from the Environmental Risk (E-Risk) Longitudinal Twin Study, which captured SIB and PEs at ages 12 and 18 years, we sought to execute the first known longitudinal, bidirectional analysis of SIB and PE occurrence. Given the extant literature on suicide occurrence/risk in the context of psychosis it was anticipated that SIB at age 18 would be predicted by the presence of PEs at age 12. It was also expected, based on the predictions of the suicidal drive hypothesis, that SIB at age 12 would significantly predict PEs at age 18. Importantly, these predicted cross-lagged associations were expected to emerge while statistically controlling for a range of risk variables, known to be associated with both phenomena and, in a more restrictive context, where children who experienced PEs at age 12 years were omitted from analyses. The current study also sought to explore the phenomenology of PEs for those who were/had engaged in SIB. Where both phenomena were reported at age 18, it was expected that PEs would contain or be characterized by SIB/threat/death-related content.

## Method

### Study cohort

Participants were members of the E-Risk Longitudinal Twin Study, which tracks the development of a nationally representative birth cohort of 2232 British twin children born in 1994–1995 and initially assessed at age 5 (93% of those eligible). The sample comprised 56% monozygotic twin pairs and 44% dizygotic (DZ) twin pairs; sex was evenly distributed within zygosity (49% male). E-Risk participants are representative of UK households across the spectrum of neighborhood socioeconomic conditions (Odgers, Caspi, Bates, Sampson, & Moffitt, 2012). Follow-up home visits were conducted when participants were aged 7, 10, 12, and 18 (participation rates were 98%, 96%, 96%, and 93%, respectively). The Joint South London and Maudsley and the Institute of Psychiatry Research Ethics Committee approved each phase of the study. Parents gave informed consent, and participants gave assent at ages 5–12 and informed consent at age 18. Full details about the sample are reported elsewhere (Moffitt, 2002) and in the Supplementary Materials.

### Measures

#### Childhood PEs

E-Risk families were visited by mental health trainees or professionals when children were aged 12. Each child was privately interviewed about seven PEs pertaining to delusions and hallucinations. Items and interviewer notes were assessed by a psychiatrist expert in schizophrenia, a psychologist expert in interviewing children, and a child and adolescent psychiatrist to verify the validity of the PEs. This interview and coding procedure have been described in detail previously (Polanczyk et al., 2010) and in the Supplementary Materials. A total of 5.9% of children reported experiencing at least one definite PE (*n* = 125). Different PE categories (hallucinations, delusions, thought interference) were also created by summing the relevant items, with a score of one or more indicating that the PE category was present.

#### Adolescent PEs

Adolescent PEs were assessed in private interviews when participants were aged 18 using the same methodology as used at age 12 (Polanczyk et al., 2010), but this time enquiring about PEs they may have experienced since age 12. Responses to the seven hallucination/delusion items were verified by a team of clinicians, including child and adolescent psychiatrists, to capture clinically pertinent PEs. Full details on the verification procedure for PEs are provided in the Supplementary Materials. At age 18, 2.9% (*n* = 59) of participants were designated as having experienced at least one definite PE between ages 12 and 18. Again, different PE categories (hallucinations, delusions, thought interference) were created by summing the relevant items, with a score of one or more indicating that the PE category was present.

#### Childhood self-injurious behaviors

Mothers were asked whether each twin had ever deliberately harmed him/herself or attempted suicide in the previous six months, as part of a face-to-face interview when the children were aged 12. Mothers who responded positively to this question were asked to provide a description of what took place. An independent rater blind to other data later checked the notes taken during the interview to verify that the description provided was clearly an act of self-harm. Only mothers, and not children, were asked to report the child’s self-harm because of ethical considerations at this age. Examples of self-injurious behaviors included cutting and biting arms, pulling out clumps of hair, banging head against walls, and attempted suicides by strangulation. At age 12, 2.9% (*n* = 62) of study members were reported to have engaged in self-injurious behaviors.

#### Adolescent self-injurious behaviors

At age 18, participants were interviewed about self-harm and suicide attempts since age 12, using a life history calendar (Caspi et al., 1996) to improve accuracy of recall. To assess self-harm, participants were asked if they had tried to hurt themselves to cope with stress or emotional pain. To assess suicide attempt, participants were asked if they had tried to kill themselves. At age 18, 13.2% (*n* = 294) of study members reported they had engaged in self-injurious behaviors.

#### Covariates

A range of covariates, known to predict both SIB and PEs (sex, childhood victimization, family history of suicide, family history of psychiatric disorders, externalizing problems and internalizing problems at age 5, and family socioeconomic status [SES]), were statistically controlled for to ensure that associations between both phenomena, at both time points, were not attributable to other common sources of risk. Details about the covariates and victimization assessments have been reported previously (Danese et al., 2017; Fisher et al., 2015) and are provided in the Supplementary Materials.

### Statistical analyses

**Figure 1.**
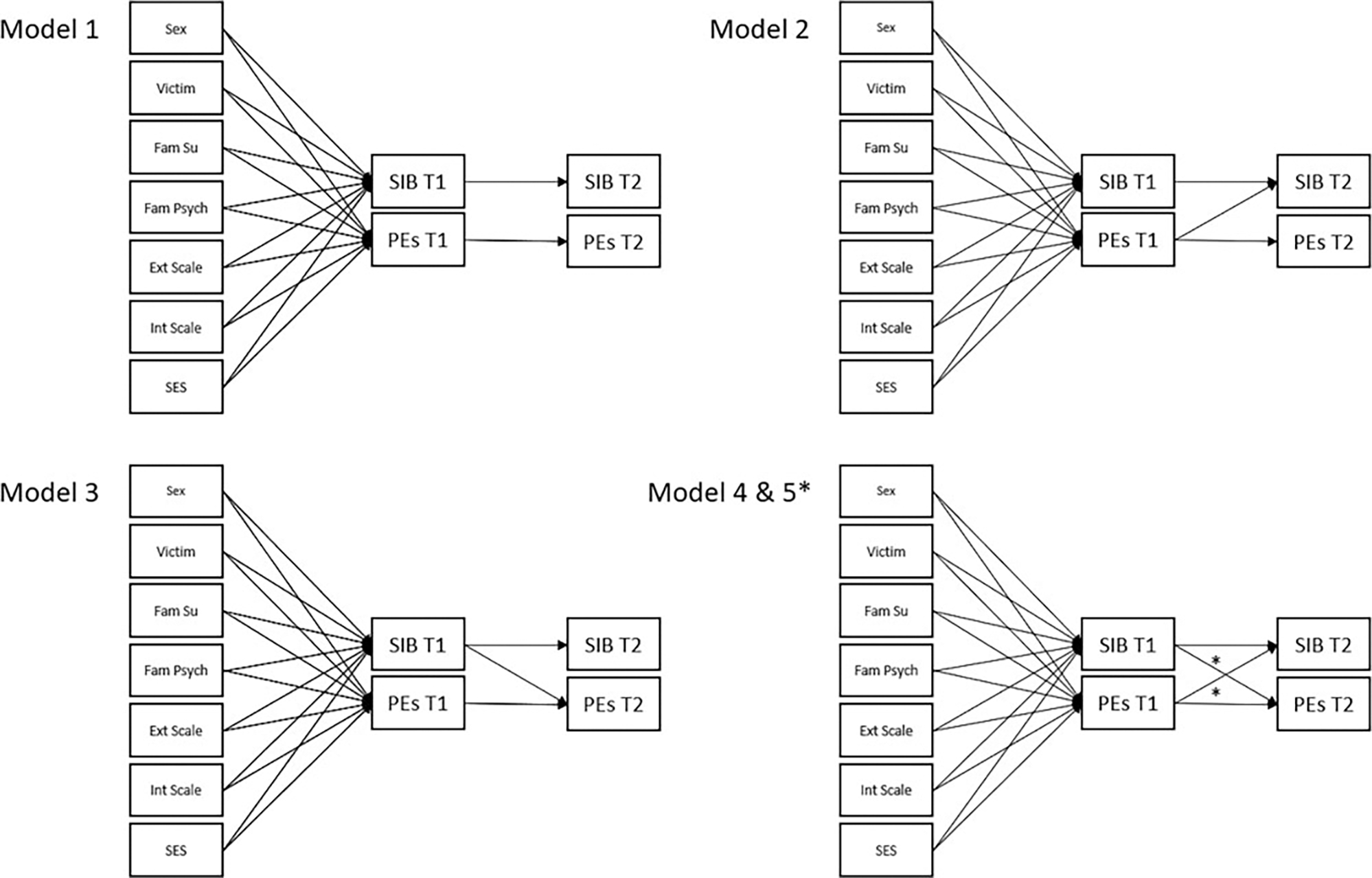
Covariate adjusted autoregressive and cross-lagged panel Models 1–5. * Cross lagged paths freely estimated in Model 4 and constrained to be equal in Model 5. Victim = childhood victimization; Fam Su = family history of suicide; Fam Psych = family history of psychiatric disorders; Ext Scale = externalizing problems; Int Scale = internalizing problems; SES = family socioeconomic status; SIB = self-injurious behavior; PEs = psychotic experiences; T1 = Time 1 (age 12 years); T2 = Time 2 (age 18 years)

Five cross-lagged models were used to test the hypotheses about the temporal relationships between PEs (measured using age-12 and age-18 clinician-verified adolescent PEs) and SIB at ages 12 and 18 years while controlling for covariates (sex, victimization, family history of SIB, family history of psychiatric disorder, internalizing and externalizing problems, and dummy coded family SES; see Figure 1). Model 1 specified only autoregressive paths for PEs and SIB; this was a baseline model specifying that all cross-lagged effects were zero. Models 2 and 3 retained the autoregressive paths but specified only unidirectional paths between PEs and SIB; Model 2 specified PEs at age 12 to predict SIB at age 18, and Model 3 specified SIB at age 12 to predict PEs at age 18. Model 4 included both cross-lagged paths, and Model 5 imposed an equality constraint on the cross-lagged paths. This fifth model tested the assumption that both phenomena were equally predictive of one another and that neither was dominant in their temporal influence.

**Figure 2.**
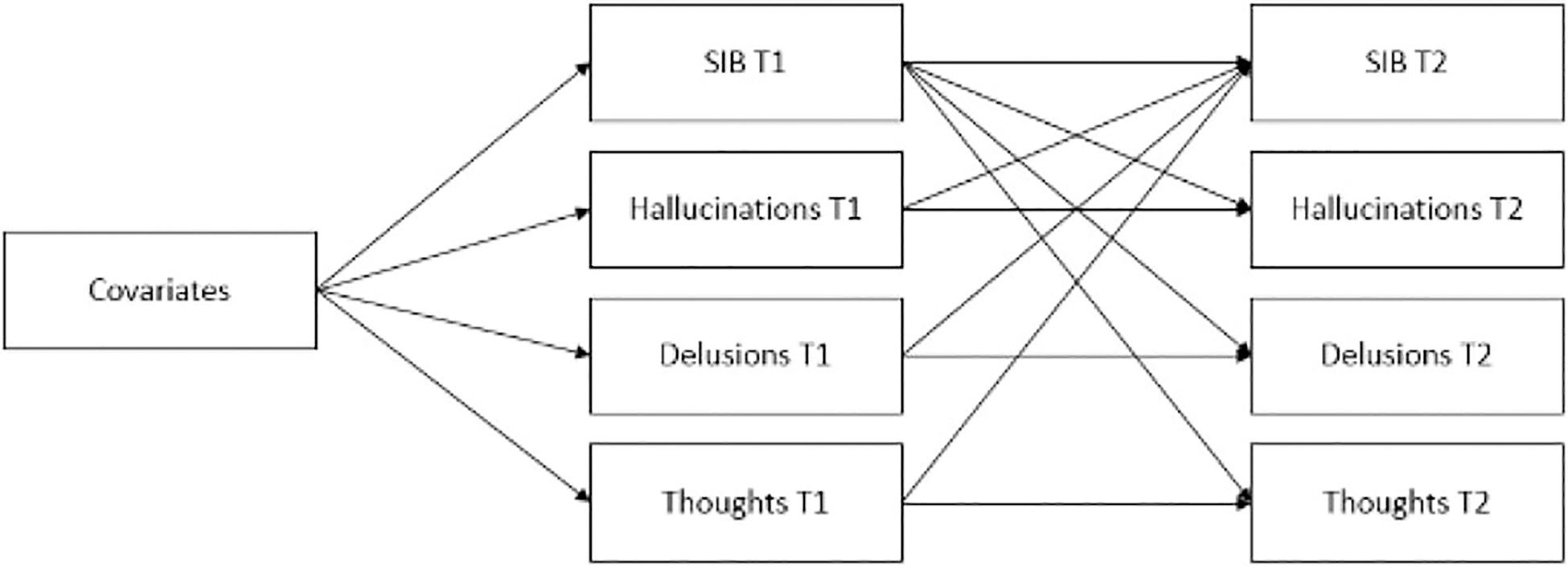
Cross-lagged effects between suicidal/self-harm behavior (SIB) and psychotic experience categories at T1 and SIB and psychotic experience categories at T2. Covariates = sex; childhood victimization; family history of suicide; family history of psychiatric disorders; externalizing problems; internalizing problems; family socioeconomic status; T1 = Time 1 (age 12 years); T2 = Time 2 (age 18 years)

Contingent on the performance of the first five models and the superior fit of models recognizing a pathway from SIB at age 12 to PEs at age 18, symptom specificity analyses (using the clinician-verified PEs at ages 12 and 18) were conducted to explore the temporal associations between PEs and SIB in more detail, where PEs were represented by three separate PE categories (hallucinations, delusions, thought interference). The model is shown in Figure 2. This model was tested to identify which PE categories were associated with SIB over time while controlling for covariates. A set of “sensitivity analyses” were also conducted to test associations between PEs (measured using (a) overall PEs and (b) separate PE categories) and SIB where PEs were absent at age 12. All models were specified and estimated using Mplus v7.0 (Muthén & Muthén, 2012) using robust maximum-likelihood estimation with the nonindependence of twin observations accounted for by using family unity as a cluster variable. For all models the PE and SIB variables were specified to be categorical and the estimates were logits, transformed to odds ratios for interpretation.

For Models 1 to 5, the best fitting model was determined using the Akaike information criterion (AIC; Akaike, 1987), the Bayesian information criterion (BIC; Schwarz, 1978), and the sample-size-adjusted BIC (ssaBIC; Sclove, 1987), with lower values indicative of better model fit. The model with the lowest BIC value was considered to be the better model and a difference of ≥10 was considered to be indicative of a “significant” difference (Raftery, 1995); if the difference between models was <10 then the model with fewer parameters would be considered the better model based on parsimony.

To test whether SIB in the absence of PEs at age 12 years was predictive of PEs at age 18 years, a more restrictive analysis was conducted on (a) the best fitting model from Figure 1 and (b) the PE category model illustrated in Figure 2. These models omitted all participants with PE at age 12 years to test whether SIB in the absence of PEs at age 12 would be predictive of PEs at age 18.

A discordant twin analysis was also conducted to rule out family-wide influences on associations between both phenomena. Specifically, we tested whether twins experiencing SIB at age 12 would be more likely to report PEs at age 18 compared to their SIB-free co-twins over and above shared familial environmental and (at least partially) genetic risks.

Finally, to explore and compare the phenomenology of PEs between those who were, or were not, dealing with a history of SIB at age 18, individual clinical interview case notes were reviewed. References to SIB were recorded, counted, and compared across both groups independently by the lead and senior authors.

## Results

Table 1 shows the frequencies and relative percentages of both phenomena at the two time points. Of those who reported SIB at age 12, 43% reported SIB again at age 18 years while 9% reported PEs at age 18. Of those with PEs at age 12, 12% reported PEs when assessed at age 18 years while 29% reported SIB at age 18. The co-occurrence of both phenomena at age 12 years was 9% and at 18 years the co-occurrence was 56%.

### Is there a bidirectional association between SIB and PEs?

The fit statistics for the competing cross-lagged models are presented in Table 2. The information criteria were all lowest for Model 5. This model returned superior fit across all fit indices. Of the two autoregressive paths (path from SIB at T1 to SIB at T2 and path from PEs at T1 to PEs at T2), PEs was the stronger (*OR* = 3.94/*OR* = 5.53, respectively) while the cross-lagged paths, that were constrained to be equal, showed that PEs at 12 years significantly increased the risk of SIB at 18 years, and SIB at 12 years significantly increased the risk of PEs at 18 years (*OR* = 2.48).

All models were also fitted with covariate paths fixed to zero. The fit indices (AIC, BIC, and ssaBIC) for these models suggested poorer fit compared to the covariate adjusted models, that is including the covariate effects resulted in improved models. The estimates of the autoregressive and cross-lagged effects in these covariate constrained models were essentially the same for the models with and without the covariates included (see Table S1 in the Supplementary Materials).

Where PEs were categorized into hallucinations, delusions, and thought interference and analyzed together with SIB at both time points, only hallucinations at age 12 predicted SIB at age 18 (*OR* = 2.36). Conversely, SIB at age 12 significantly predicted both hallucinations (*OR* = 2.77) and thought interference symptoms (*OR* = 4.15) at age 18 (see Table 3).

Where models excluded cases with any PEs at age 12, no significant association was found between SIB at age 12 years and PEs at age 18 years (*OR* = 1.06; 95% CI = 0.14–7.94). However, where the three PE categories were modeled (i.e., Figure 2 model excluding cases with Hallucinations T1, Delusions T1, and Thought interference T1), effects for hallucinations (*OR* = 2.00; 95% CI = 0.72–5.59) and delusions (*OR* = .67; 95% CI = 0.09–4.98) were nonsignificant, but the association between SIB at age 12 years and thought interference at age 18 years was strong (*OR* = 4.29) and trended towards significance (95% CI = 0.97–19.01).

### Does the prospective association between SIB and PEs hold when controlling for unmeasured familial environmental and (at least partially) genetic confounding?

In total, 52 twin pairs were discordant for SIB at age 12. Twins who experienced SIB at this age were significantly more likely to report PEs at age 18 compared to their co-twins who did not experience SIB (7 (28.0%) versus 2 (7.4%); ratio = 2.78, 95% CI 1.55 to 4.98).

### Are PEs among those experiencing SIB informed and characterized by SIB/threat/death-related content?

Upon reviewing the clinical interview case notes for those who reported both SIB and PEs at age 18 (*n* = 33), PEs for 39% of cases were explicitly characterized and informed by SIB/threat/death-related themes/content. For those who only reported PEs at age 18 (*n* = 26), just one individual’s PEs (4%) were characterized by SIB/threat/death-related themes/content (see content, descriptions, and interpretations of adolescent PEs in the Discussion and Supplementary Materials).

## Discussion

### SIB – PEs directionality

The findings from the cross-lagged panel analyses were inconclusive. An overall model that constrained both paths to be equal (i.e., temporal associations from (a) PEs to SIB and (b) SIB to PEs) returned the best fit. This model (Model 5) demonstrated that neither variable at age 12 was dominant in predicting the other at age 18, but instead that each variable increased the probability of the other, across a six-year assessment period, by approximately two-and-a-half times. While it was notable that the second best fitting model (Model 2) was an explicit test of the orthodox view (i.e., where SIB is consequential to PEs but PEs are not recognized in a predictive capacity), the freely estimated model (Model 4; where coefficients for all paths were estimated) demonstrated that the strongest cross-lagged path was between SIB at age 12 and PEs at age 18 (*OR* = 2.82).

Once it was established that SIB was predictive of PEs in the overall model, it was necessary to consider which PE categories (hallucinations, delusions, thought interference) were predicted by earlier SIB, and how strongly. The PE category cross-lagged model demonstrated that those who reported SIB at age 12 were over 2.5 times more likely to report hallucinatory experiences and over 4 times more likely to report thought interference at age 18.

No association, however, was identified between SIB and the delusions symptom category. Given that psychosis-related paranoia and persecutory delusions often include beliefs about personal safety and impending danger (Bentall, Kinderman, & Kaney, 1994; Freeman et al., 2007), this was unexpected. However, psychotic delusions are not always colored by threat (e.g., grandiose delusions); therefore, the lack of specificity and detail regarding the precise nature and phenomenology of delusional experience in this variable may not have afforded the most robust test of this association in a suicidal drive context. It was notable that where phenomenology was recorded (see case note content in following paragraphs), delusions among 18-year-olds reporting SIB did include paranoia and persecutory/referential delusions that contained SIB or death-related themes/content.

The more restrictive sensitivity analyses that omitted those without PEs at age 12 years revealed that SIB, in the absence of PEs, did not predict overall PEs at age 18 years but may have been predictive of thought interference. These findings firstly suggested that the influence of SIB at age 12 years on later overall PEs (as evidenced in Model 5) was likely attributable to co-occurring PEs at age 12. Given the proposed role of psychosis in the context of SIB, as purported by the suicidal drive hypothesis, this was not entirely surprising. Of the 125 children who experienced PEs and the 62 who experienced SIB at age 12 years, 11 experienced both phenomena. Of these, 91% (*n* = 10) experienced SIB at age 18. This group therefore exhibited the greatest risk for SIB at age 18 (compared to 31% for those only experiencing SIB at age 12 years and 13% for those who displayed no PEs or SIB at this age) which in turn would plausibly explain “defensive” PE expression at this later age. Removing these children from the analysis therefore essentially removed those at greatest risk of later SIB and, in so doing, removed those for whom a protective PE reaction would have been most likely. Secondly, the potential effect (i.e., 95% CI = 0.97–19.01) of SIB only at age 12 years on thought interference at age 18 years suggested that where SIB was not accompanied by PEs early on, it may have acted on a specific PE category. The young age of the sample (at both time points), the low occurrence of SIB among the sample at age 12 years, and the six-year lag between assessment time points, however, limited the interrogation of both phenomena.

In summary, therefore, while SIB was initially shown to be predictive of later PEs, this was no longer the case once the effect of individuals with preexisting PEs was removed. Moreover, at a PE category level, where SIB was initially shown to be predictive of later hallucinatory experiences and thought interference, SIB in the absence of PEs at age 12, was not predictive of PE categories at age 18; however, there was a (nonsignificant) trend for SIB at age 12 years to predict later thought interference.

### The phenomenology of PEs in the context of SIB

The clinical interview case notes of all adolescents who reported both PEs and SIB at age 18 (*n* = 33) revealed 13 individuals (39%) whose PEs were characterized by SIB/threat/death-related themes/content. The most commonly reported PEs were auditory verbal hallucinations, followed by intrusive thoughts, paranoia, and persecutory/referential delusions.

### Auditory verbal hallucinations

A total of 9 out of the 13 adolescents (69%) described auditory verbal hallucinations characterized by commands, instructing them to kill, harm, or hurt themselves. While details about these hallucinatory experiences, beyond the general SIB instruction, were limited in some case notes, others offered notable descriptions of the auditory verbal hallucinations and in turn revealed important variation in the nature of these experiences. For example, some commands were explicit and contained precise instructions regarding suicidal behavior. For example; (a) “Go into the middle of the road, someone will hit you”; (b) “The twin stated the voices have made her think about overdosing previously”; (c) “For instance, when she was going to jump off [name of structure removed to preserve anonymity] the voice was telling her to do it”; (d) “Twin said she would hear voices when self-harming, and that they would egg her on, encouraging her to cut herself nearer to veins and to kill herself”; (e) “The man [voice] would say mean things to the twin such as ‘you’re not worth anything’ and ‘there’s a red knife in the kitchen”. The extant research literature is replete with studies that have explored command hallucinations in the context of clinical psychosis and SIB (Harkavy-Friedman et al., 2003; Rogers, Watt, Gray, MacCulloch, & Gournay, 2002; Zisook, Byrd, Kuck, & Jeste, 1995); however, there has been limited explanation for the co-occurrence of these phenomena (Bolton, Gooding, Kapur, Barrowclough, & Tarrier, 2007; Johnson, Gooding, & Tarrier, 2008; Taylor et al., 2010). The suicidal drive hypothesis potentially affords an opportunity to begin to consider these voices as external attributions of internal threat. It may be the case that under circumstances of internally generated and self-directed threat, individuals attribute this threat (albeit unconsciously) to sources other than, and external to, themselves in order to secure some psychological distance/protection. Given that this distance/protection can only manifest psychologically, internal suicidal/self-injurious thoughts may ultimately become external commands/instructions, communicated by external agents/voices.

### Intrusive thoughts, paranoia, and persecutory/referential delusions

A total of 6 of the 13 adolescents’ PEs (46%) were characterized by intrusive thoughts, paranoia, and persecutory/referential delusions that contained SIB or death-related themes/content. Two adolescents believed that murders/killings reported on the news were attributable to them, while others revealed paranoia and worry about being hurt, poisoned, or attacked (a) “Twin believes that when people on the news report about murders and missing people they are talking about her”; (b) “Twin answered yes saying that for example he sometimes gets a glass of water (whilst at home) & thinks that it doesn’t taste right, which will get him worried that it might be poisoned”. Two adolescents described their symptoms specifically in relation to eating-related SIB, for example (a) “[name removed to preserve anonymity] – the name/identity she gave to her anorexic thoughts….She said he was ‘higher than her’, and had control/power over her….Punishment came when he didn’t get what he wanted – i.e., wasn’t skinny/able to see bones. Punishment would be having to make herself sick if she ate something not ‘allowed’”; and (b) “They [voices] were calling the twin fat and saying she should not eat”. While auditory verbal hallucinations, particularly those characterized by explicit suicide instruction, afford the clearest description of what the suicidal drive hypothesis posits, the hypothesis also considers other PEs to be meaningful manifestations of internal threat. It seems plausible that if internal threat is externalized, it may be observable also in delusional ideation and paranoia about, for example the malevolent intentions of others to cause harm or the general/specific presence of threat in one’s environment.

### Disowned aspects of self and negative self-evaluation

The suicidal drive hypothesis proposes that in the context of SIB, individuals may attribute their internal threat to sources other than, and external to, themselves in order to secure some psychological distance/protection. This protective distancing from thoughts/beliefs that have become threat laden, therefore, may lead to confusion about, for example one’s sense of agency and/or ownership of thought which may also be evident in psychosis symptom expression/content. Some of the case notes made reference to an inner turmoil experienced by the adolescents; for example, (a) “the twin feels threatened, he feels like part of him is telling him to act one way whereas another part is telling him to do the opposite” and (b) “Says [voice] will make her feel bad about herself when she feels positive – so if she thinks she looks nice it would say she looks awful – telling her no one likes her.” This is largely consistent with what has been observed elsewhere in phenomenological analyses of auditory verbal hallucinations in psychosis. For example, in a qualitative study of clinical voice hearers, it was possible to clearly formulate the underlying emotional conflicts embodied by the voices (e.g., low self-worth) in 94% of cases, while representations for voice identity (e.g., disowned aspects of self) were formulated in 78% of cases (*N* = 100; Corstens & Longden, 2013). Moreover, while suicidality may constitute the most severe form of internal threat, it is possible that psychosis may also be consequential to less severe thoughts and beliefs that ultimately fuel suicidality, for example low self-esteem, feelings of inadequacy, self-criticism, shame, submissive behavior, self-disgust, and so on (Butter, Shevlin, & Murphy, 2019). It was notable also that several adolescents’ case notes referred to feelings of worthlessness and low self-esteem. For example: (a) “The voice insults the twin, saying that she is worthless, it wasn’t always insulting but as her self-esteem decreased as did the demon’s belief of her”, and (b) “When the voice would give a command, it would also make derogatory comments such as ‘you are not worth being here’ and ‘you are stupid’”. Bentall and colleagues have demonstrated that feelings of inferiority and inadequacy (known to be central to SIB) may underlie many positive psychotic symptoms and may ultimately influence their distressing content and persistence (Bentall, Corcoran, Howard, Blackwood, & Kinderman, 2001). Indeed, changes in self-esteem over time have been associated with fluctuations in symptom severity while a disturbed self-esteem has been implicated in maintaining florid paranoid ideation (Garety et al., 2005; Häfner et al., 2005; Thewissen, Bentall, Lecomte, van Os, & Myin-Germeys, 2008).

### Research implications

The current study is the second to explore the suicidal drive hypothesis. While the evidence produced thus far seems to offer some tentative support for the proposed hypotheses (albeit limited to the temporal associations between the phenomena and the phenomenology of psychosis symptomology), the findings will need to be replicated and a more stable empirical foundation established before the protective/defense assumptions of the suicidal drive hypothesis can be justifiably explored. At present, however, it is worth considering how such assumptions regarding defense and protection might be tested. At a rudimentary level we might expect that suicide attempts for those who both engage in suicidal ideation/SIB and experience PEs will be delayed or prevented compared to those who experience suicidal ideation/SIB in the absence of PEs. We might also expect, at “less severe” levels, that the occurrence of SIB is delayed or prevented for those engaged in or enduring suicidal ideation. Determining when/if PEs emerge, however, along this suicidality continuum (i.e., after suicidal ideation, SIB or suicide attempts) will be important.

Future research might also consider whether the suicidal drive hypothesis can be meaningfully employed to describe other related phenomena in the literature. Comorbidity, for example, between psychotic disorders and suicide-informed diagnoses such as major depression, borderline personality disorder, and eating disorder (Birchwood, Iqbal, Chadwick, & Trower, 2000; Blinder, Cumella, & Sanathara, 2006; Kingdon et al., 2010) may reflect the suicidal drive – psychosis threat response in action. Selten and Cantor-Graae’s social defeat hypothesis of schizophrenia (Selten, van der Ven, Rutten, & Cantor-Graae, 2013) and Taylor et al.’s review of the role of both social defeat and entrapment for a wide array of psychopathological phenomena would also seem to align well with the assumptions that are proposed by the suicidal drive hypothesis (Taylor, Gooding, Wood, & Tarrier, 2011). Moreover, the extensive childhood trauma – PE literature (Badour & Adams, 2015; Read, van Os, Morrison, & Ross, 2015; Schalinski & Teicher, 2015) may be informed by an internal threat response hypothesis, in that childhood trauma (particularly interpersonal and sexual childhood trauma) has been shown to be highly associated with “mental contamination” (Badour & Adams, 2015), self-denigration (Gilbert, 2015), self-disgust (Overton, Powell, & Simpson, 2015), self-harm (Saçarçelik, Türkcan, Güveli, & Yeşilbaş, 2015), and SIB (Park, Hong, Jeon, Seong, & Cho, 2015). PEs among childhood trauma victims therefore, while reflective of trauma response, may no longer directly reflect response to the original external traumatic event but instead may reflect defense from the internally generated and self-directed threat derived from the trauma-induced shame, guilt, depression, and disgust. Importantly, however, the current findings offer only preliminary empirical support for an internal threat response hypothesis. Further analyses will be required to substantiate the speculative assumptions applied to the wider literature briefly outlined here.

### Clinical implications

While it is recognized that the suicidal drive hypothesis will need to be evidenced more fully before clinical implications can be realistically explored, two potential clinically related issues can be tentatively considered and shared here. First, the suicidal drive hypothesis may have some utility in informing extant interventions for those who experience PEs in the context of suicidality. For example, recognizing voices as disowned aspects of oneself, and dialoguing with/challenging voices has become increasingly popular in treatment settings for distressed voice hearers (Craig et al., 2018; Deamer & Hayward, 2018; Leff, Williams, Huckvale, Arbuthnot, & Leff, 2014; Lincoln & Peters, 2019; Thomas et al., 2014). Given that the suicidal drive hypothesis recognizes auditory verbal hallucinations to be audible manifestations of internal, self-directed threatening thoughts/beliefs, clinicians may consider treatment strategies that directly target self-threat vulnerability and negative self-evaluation among those traumatized by auditory verbal hallucinations. Inviting voice hearers to consider, acknowledge, and attend to the presence of suicidal thoughts and beliefs may in turn (a) afford them an opportunity to recognize the inherent danger that they have become exposed/vulnerable to, and (b) consider (and potentially understand) why such thoughts and beliefs have been “disowned”, attributed to external sources, and perceived as voices from without. Second, the suicidal drive hypothesis may also shed light on why delusional beliefs are often so resilient in the presence of contradictory evidence, and why those that hold them are so resistant to the challenges/perspectives of others (Freeman, Garety, & Kuipers, 2001; Garety et al., 2005). While researchers have previously proposed safety seeking/behavior explanations for psychotic delusions (Freeman et al., 2001; Freeman et al., 2007), according to the suicidal drive hypothesis, delusional beliefs (particularly threat-laden persecutory/referential delusions in the context of SIB) serve to externalize internal threat, and attribute it to external agents/sources. This external attribution, therefore, may also be conceived to be protective and may afford “distance” (psychological distance) from a threat that ultimately resides within. Investment in and commitment to these beliefs/schemas, therefore, may be necessary for survival, and therapists and clients alike may need to recognize this before either phenomenon (i.e., suicidality or psychosis) can be successfully navigated or treated.

### Study limitations

While the current study produced additional empirical support for the suicidal drive hypothesis it was not without its limitations. First, the analyses were not inclusive of suicidal ideation. Suicidal ideation has been shown to be highly predictive of both SIB and suicide attempts and has previously been conceptualized as a constituent “component” of a proposed suicidality continuum (Butter et al., 2019; Klonsky, May, & Saffer, 2016; Ribeiro et al., 2016). While its absence in the current study was unlikely to have negated the suicidal drive pathway identified (i.e., supplementing the SIB variable with suicidal ideation would likely have amplified this association) its inclusion and exploration in future prospective analyses will be important. Second, self-injurious and suicidal behavior were aggregated to reflect overall internal threat in the current study. These behaviors have, however, been shown to be distinct from one another (Halicka & Kiejna, 2015) and should be tested separately in future studies where larger/older sample data and higher prevalence rates facilitate such analyses. Third, SIB at 12 years was informed by parental report while PEs at this age were derived from child self-reports. It is possible that there may have been some false negatives for SIB at this time point. Fourth, the current cross-lagged panel analyses, although sophisticated and suitable, were based on data from only two time points, with a 6-year time-lag. In previous Danish prospective cohort analyses of the suicidal drive hypothesis, it was notable that most recorded clinical occurrences of SIB and psychosis emerged in the same year (Murphy et al., 2018). Moreover, the current data captured both SIB and PEs within a highly sensitive developmental age range where the meaning and experience of both phenomena may have varied substantially. To more robustly test the suicidal drive hypothesis, future analyses will need to test temporal associations between both phenomena over multiple, shorter lagged, time points. Fifth, while SIB informed PEs were more commonly identified for those who reported both SIB and PEs at age 18 (39%), compared to those who only reported PEs (4%), PEs for most adolescents reporting both phenomena at age 18 were not characterized by SIB content. While it is possible that the PEs reported by these adolescents were associated with their SIB (i.e., it may be plausible to consider nonthreatening grandiose or referential delusions also in terms of suicidal drive hypothesis threat responsivity), descriptions of their experiences did not reveal evidence of, for example, threat perception/belief, preoccupation with death, or SIB instruction. Sixth, it is possible that some of the twins at age 18 may have been in an extended prodrome and their SIB at age 12 years was a manifestation of their distress. Seventh, alternative molecular and neural substrates of SIB known to influence the development of stable emotional, behavioral, and cognitive phenotypes (Balcioglu & Kose, 2018; Turecki, 2014; Turecki, Ernst, Jollant, Labonté, & Mechawar, 2012) were not controlled for and may afford alternative explanations for the findings reported. Eighth, the E-Risk study included data from twins only; therefore, we cannot be totally sure that these findings can generalize to non-twins. However, the prevalence of SIB and PEs reported here are similar to those observed in singletons (Horwood et al., 2008; Kidger, Heron, Lewis, Evans, & Gunnell, 2012). Ninth, the sample size used in the current study was relatively small. Larger samples will afford more robust tests of the proposed associations evidenced here.

## Conclusion

The suicidal drive hypothesis has now been partially evidenced by findings from analyses of cross-sectional epidemiological data, national prospective cohort/service use data, and, in this study, prospective twin cohort data. Collectively these analyses have tentatively demonstrated that (a) suicidality (ranging from suicidal ideation to self-injurious behavior and suicide attempts) is highly associated with psychosis (in both clinical and subclinical contexts); (b) suicidality can precede psychotic phenomena; (c) the strength of the association between PEs and SIB varies according to both SIB recency and severity; and (d) PEs among those enduring SIB are often informed and characterized by SIB content. Researchers in future therefore should be cognizant of this bidirectional association and be mindful that where SIB is present among those experiencing PEs, SIB may not necessarily be consequential to PEs. Researchers and practitioners alike might also begin to consider the potential protective/defensive role of PEs in a context of SIB and explore why PEs are (a) so commonly characterized by threat-related content, and (b) experienced as external to or separate from the self. Finally, practitioners should be alert to psychosis risk among those presenting with SIB (and vice versa) and consider interventions that might help prevent individuals from developing even more complex mental health problems.

## Supplementary Material

supplemental material

**Supplementary Material.** The supplementary material for this article can be found at https://doi.org/10.1017/S0954579420001728.

## Figures and Tables

**Table 1.** Cross-tabulations of suicidal/self-harm behavior (SIB) and psychotic experiences (PEs) within and across time points (*N* = 2232^a^)

	SIB T2		PEs T2		SIB T1	
	Present, *n* (%)	Absent, *n* (%)	Present, *n* (%)	Absent, *n* (%)	Present, *n* (%)	Absent, *n* (%)
SIB T1						
Present	24 (42.9)	32 (57.1)	5 (8.9)	51 (91.1)	—	—
Absent	261 (13.4)	1,693 (86.6)	51 (2.6)	1,903 (97.4)	—	—
PEs T1						
Present	34 (29.1)	83 (70.9)	14 (11.9)	104 (88.1)	11 (8.8)	114 (91.2)
Absent	248 (13.1)	1,639 (86.9)	42 (2.2)	1,847 (97.8)	51 (2.6)	1,947 (97.4)
PEs T2						
Present	33 (55.9)	26 (44.1)	—	—	—	—
Absent	259 (12.9)	1,742 (87.1)	—	—	—	—

a Missing data for 109–228 cases across cells; Proportions correspond to within row variable. T1 – age 12 years; T2 – age 18 years.

**Table 2.** Associations between suicidal/self-harm behavior (SIB) and psychotic experiences (PEs) at ages 12 (T1) and 18 (T2) years

	Model 1 Baseline	Model 2 PEs → SIB	Model 3 SIB → PEs	Model 4 Bi-directional Free	Model 5 Bi-directional Constrained
Model fit					
AIC	3,432.130	3,419.958	3,430.826	3,418.656	3,416.726
BIC	3,556.590	3,550.076	3,560.944	3,554.431	3,546.844
ssaBIC	3,486.694	3,477.002	3,487.870	3,478.180	3,473.770
Autoregressive paths (*ORs* (95% CIs))					
SIB T2 on SIB T1	4.42 (2.53–7.75)	3.95 (2.34–6.67)	4.42 (2.53–7.75)	3.95 (2.34–6.67)	3.94 (2.35–6.62)
PEs T2 on PEs T1	5.94 (3.05–11.59)	5.95 (3.05–11.59)	5.45 (2.81–10.58)	5.46 (2.81–10.59)	5.53 (2.87–10.64)
Cross-lagged paths (*ORs* (95% CIs))					
SIB T2 on PEs T1	—	2.43 (1.54–3.82)	—	2.43 (1.54–3.82)	2.48 (1.63–3.79)
PEs T2 on SIB T1	—	—	2.82 (1.15–6.92)	2.82 (1.15–6.92)	2.48 (1.63–3.79)

AIC = Akaike information criterion; BIC = Bayesian information criterion; CIs = confidence intervals; ORs = odds ratios; ssaBIC = sample-size adjusted BIC. All models statistically adjusted for the nonindependence of twin observations and covariates = sex; childhood victimization; family history of suicide; family history of psychiatric disorders; externalizing problems; internalizing problems; family socioeconomic status.

**Table 3.** Autoregressive and cross-lagged effects between suicidal/self-harm behavior (SIB) and psychotic experience categories at ages 12 (T1) and 18 (T2) years

	*ORs* (95% CIs)			
	SIB T2	Hallucinations T2	Delusions T2	Thought interference T2
SIB T1	**3.99** (2.34–6.80)	**2.77** (1.27–6.03)	1.65 (0.60–4.53)	**4.15** (1.29–13.37)
Hallucinations T1	**2.36** (1.70–3.27)	**4.36** (2.92–6.52)	—	—
Delusions T1	1.08 (0.64–1.82)	—	**3.33** (1.79–6.19)	—
Thought interference T1	1.09 (0.47–2.54)	—	—	**13.61** (5.18–35.72)

Statistically significant paths at *P* < .05 in bold; CIs = confidence intervals; *ORs* = odds ratios; Models adjusted for the nonindependence of twin observations and covariates = sex; childhood victimization; family history of suicide; family history of psychiatric disorders; externalizing problems; internalizing problems; family socioeconomic status.

## Footnotes

1 As of 22/11/2019.
