# Supplementary material for: Externalizing the threat from within: A new direction for researching associations between suicide and psychotic experiences
Source: Dev Psychopathol. Author manuscript; Available in PMC 2026 Mar 23. (PMC13007553; doi:10.1017/S0954579420001728)
Supplement: supplemental material [file NIHMS2146080-supplement-supplemental_material.doc]

**Supplementary Materials**

*Murphy et al. “Externalising the threat from within: A new direction for researching associations between suicide and psychotic experiences.”*

**Study Cohort**

Participants were members of the Environmental Risk (E-Risk) Longitudinal Twin Study, which tracks the development of a nationally-representative birth cohort of 2232 British twin children. The sample was drawn from a larger cohort of twins born in England and Wales in 1994-1995 (Trouton et al., 2002). Full details about the sample are reported elsewhere (Moffitt & E-Risk Study Team, 2002). Briefly, the E-Risk sample was constructed in 1999-2000, when 1116 families with same-sex 5-year-old twins (93% of those eligible) participated in home-visit assessments. Families were recruited to represent the UK population of families with newborns in the 1990s, based on residential location throughout England and Wales and mothers’ age. Teenaged mothers with twins were over-selected to replace high-risk families who were selectively lost to the register through non-response. Older mothers having twins via assisted reproduction were under-selected to avoid an excess of well-educated older mothers. E-Risk families are representative of UK households across the spectrum of neighborhood-level deprivation: 25.6% of E-Risk families live in “wealthy achiever” neighborhoods compared to 25.3% of households nation-wide; 5.3% vs 11.6% live in “urban prosperity” neighborhoods; 29.6% vs 26.9% live in “comfortably off” neighborhoods; 13.4% vs 13.9% live in “moderate means” neighborhoods; and 26.1% vs 20.7% live in “hard-pressed” neighborhoods (CACI, 2006; Odgers et al., 2012). E-Risk families under-represent “urban prosperity” neighborhoods because such households are likely to be childless. The sample comprised 56% monozygotic and 44% dizygotic twin pairs, and sex was evenly distributed within zygosity (49% male). All families were English speaking, and the majority (93.7%) were White.

Follow-up home visits were conducted when children were 7 years (98% of the 1116 E-Risk Study families participated), 10 years (96% participation), 12 years (96% participation) and 18 years (93% participation). Home visits at ages 5, 7, 10, and 12 years included assessments with participants as well as their mother (or primary caretaker); the home visit at age 18 included interviews only with the participants. Each twin participant was assessed by a different interviewer. The average age of the twins at the time of the age 18 assessment was 18.4 years (*SD*=0.36); all interviews were conducted after the 18^th^ birthday. There were no differences between those who did and did not take part at age 18 in terms of socioeconomic status (SES) assessed when the cohort was initially defined (χ^2^=0.86, *p*=0.65), age-5 IQ scores (*t*=0.98, *p*=0.33), or age-5 internalizing or externalizing behavior problems (*t*=0.40, *p*=0.69 and *t*=0.41, *p*=0.68, respectively). The Joint South London and Maudsley and the Institute of Psychiatry Research Ethics Committee approved each phase of the study. Parents gave informed consent, and participants gave assent at ages 5-12 and informed consent at age 18.

**Measure of psychotic experiences (PEs)**

E-Risk families were visited by mental health trainees or professionals when children were aged 12 (Polanczyk et al., 2010). Each child was privately interviewed about 7 PEs pertaining to delusions and hallucinations, with the following items: “Have other people ever read your thoughts?” [thought interference], “Have you ever thought you were being followed or spied on?” [delusions], “Have you ever heard voices that other people cannot hear?” [hallucinations], “Have you ever believed that you were being sent special messages through the television or radio, or that a programme has been arranged just for you alone?” [thought interference], “Have you ever felt like you were under the control of some special power?” [delusions], “Have you ever known what another person was thinking, like you could read their mind?” [delusions], and “Have you ever seen something or someone that other people could not see?” [hallucinations]. This interview has been described in detail previously (Polanczyk et al., 2010). The item choice was guided by the Dunedin Study’s age-11 interview protocol (Poulton et al., 2000) and an instrument prepared for the Avon Longitudinal Study of Parents and Children (Schreier et al., 2009). Interviewers coded each experience 0, 1, 2 indicating respectively “not a symptom,” “probable symptom,” and “definite symptom.” A conservative approach was taken in designating a child’s report as a symptom. First, the interviewer probed using standard prompts designed to discriminate between experiences that were plausible (e.g., “I was followed by a man after school”) and potential symptoms (e.g., “I was followed by an angel who guards my spirit”), and wrote down the child’s narrative description of the experience. Second, items and interviewer notes were assessed by a psychiatrist expert in schizophrenia, a psychologist expert in interviewing children, and a child and adolescent psychiatrist to verify the validity of the symptoms. Third, because children were twins, experiences limited to the twin relationship (e.g., “My twin and I often know what each other are thinking”) were coded as “not a symptom”. Children were only designated as experiencing PEs if they reported at least one definite experience. At age 12, 5.9% (*N*=125) of children reported experiencing at least one definite PE. This is similar to the prevalence of PEs in other community samples of children and adolescents (Kelleher et al., 2012; Horwood et al., 2008; Yoshizumi et al., 2004; Scott et al., 2006; Dhossche et al., 2002). Furthermore, we have previously shown that childhood PEs in this cohort have good construct validity, sharing many of the genetic, social, neurodevelopmental, and behavioral risk factors and correlates as adult schizophrenia (Polanczyk et al., 2010). Different PE categories (hallucinations, delusions, thought interference) were also created by summing the relevant items, with a score of one or more indicating that the symptom category was present.

The same items and clinical verification procedure were used when participants were interviewed at age 18, this time enquiring about PEs they may have experienced since age 12. At age 18, 2.9% (*N*=59) of participants reported experiencing at least one definite PE since age 12 that was clinically verified (referred to as adolescent PEs). These rates are similar to those reported for community samples of children and adolescents in other studies using clinical verification procedures (Horwood et al., 2008; Dhossche et al., 2002). The comparatively low prevalence of PEs at age 18 versus age 12 is also consistent with findings from other studies showing an attenuating rate of PEs from childhood to adulthood (Kelleher et al., 2012; Zammit et al., 2013). Again, different PE categories (hallucinations, delusions, thought interference) were created by summing the relevant items, with a score of one or more indicating that the PE category was present.

**Covariates**

A range of covariates, known to predict both self-injurious behaviours (SIB) and PEs, were statistically controlled for to ensure that associations between both phenomena, at both time-points, were not attributable to other sources of risk. *Family socioeconomic status* (SES), commonly evidenced as an environmental risk factor for both SIB (Qin et al., 2003) and PEs (Werner et al., 2007), was measured via a composite of parental income, education, and occupation when participants were aged 5. This variable was categorised into tertiles (i.e., low-, medium-, and high-SES; Trzesniewski et al., 2006) and recoded as 2 dummy variables with ‘low SES’ as the reference category. *Family psychiatric history,* also widely recognised in aetiological investigations of both SIB (Qin et al., 2002) and PEs (Seidman et al., 2010), was assessed when participants were aged 12. In private interviews, mothers reported on family history of DSM disorders (Weissman, 2000), which was converted to a proportion (0-1.0) of family members with a history of psychiatric disorder (Milne et al., 2008). *Family history of suicide,* a strong indicator of SIB (Franklin et al., 2017) and PEs (Mortensen et al., 2010) risk, was also controlled for in this study. We determined attempted or completed suicide by any of the child’s biological mother, father, grandparents, aunts, or uncles from reports by biological parents, according to a standardised and validated protocol (Milne et al., 2009). We calculated a modified Reed’s score, which takes into account the number of affected relatives given the size and demographic structure of the family (Milne et al., 2008), and we considered children whose score was at or above the 80th centile to have a strong positive family history of suicide. *Internalising and externalising problems,* while commonly associated with a wide array of general psychopathological phenomena, have been repeatedly identified as risk factors for both SIB (Bettes & Walker, 1986) and psychotic phenomena (Downs et al., 2013). We assessed internalising and externalising problems at age 5 by using the Child Behaviour Checklist in face-to-face interviews with mothers and the teacher’s report form by mail for teachers (Achenbach, 1991a; 1991b). The internalising problems scale is the sum of items in the withdrawn and anxious/depressed subscales, and the externalising problems scale is the sum of items from the aggressive and delinquent behaviour subscales. We summed and standardised mothers’ and teachers’ reports to create cross-informant scales. Childhood victimisation is one of the most commonly investigated environmental risk factors for both SIB (Van der Kolk et al., 1991; Nock, 2009) and PEs (Schäfer & Fisher, 2011; Varese et al., 2012) and was an important covariate to adjust for in the current set of analyses. Exposure to several types of *victimisation* was assessed repeatedly when the children were 5, 7, 10, and 12 years of age and dossiers have been compiled for each child with cumulative information about exposure to domestic violence between the mother and her partner, frequent bullying by peers, physical maltreatment by an adult, sexual abuse, emotional abuse and neglect, and physical neglect. Each form of victimisation was rated by coders as “0” not present; “1” probable harm, occasionally present, or evidence of only minor incidents; or “2” definite harm, frequently present, or evidence of severe incidents. Exposure to any severe victimisation was defined as experiencing one or more types of victimisation that were coded as “2” before age 12: 73.5% of children had zero victimization experiences; 26.5% had 1 or more severe victimization experiences. Details about the victimisation assessments and evidence on the reliability and validity of them have been reported previously (Arseneault et al., 2006; Danese et al., 2017; Fisher et al., 2015; Jaffee et al., 2004; Moffitt et al., 1997; Shakoor et al., 2011). All the component measures are outlined briefly below.

*Physical Domestic Violence.* Mothers reported about perpetration of and victimization by 12 forms of physical violence (e.g., slapping, hitting, kicking, strangling) from the Conflict Tactics Scale (Straus, 1990), on three assessment occasions during the child's first decade of life (when the children were 5, 7, and 10 years of age). Reports of either perpetration or victimization constituted evidence of physical domestic violence. Families in which no physical violence took place were coded as 0 (55.2%); families in which physical violence took place on one occasion were coded as 1 (28.0%); and families in which physical violence took place on multiple occasions were coded as 2 (16.8%).

*Bullying by Peers.* Experiences of victimization by bullies were assessed using both mothers’ and children’s reports. During the interview, the following standard definition of bullying was read out: *“Someone is being bullied when another child (a) says mean and hurtful things, makes fun, or calls a person mean and hurtful names; (b) completely ignores or excludes someone from their group of friends or leaves them out on purpose; (c) hits, kicks, or shoves a person, or locks them in a room; (d) tells lies or spreads rumours about them; and (e) other hurtful things like these. We call it bullying when these things happen often, and when it is difficult to make it stop. We do not call it bullying when it is done in a friendly or playful way.”* Mothers were interviewed when children were 7, 10, and 12 years old and asked whether either twin had been bullied by another child, responding never, yes, or frequently. We combined mothers’ reports when children were age 7 and 10 to derive a measure of victimization during primary school. Mothers’ reports when the children were 12 years old indexed victimization during secondary school. During private interviews with the children when they were 12 years old, the children indicated whether they had been bullied by another child during primary or secondary school. When a mother or a child reported victimization, the interviewer asked them to describe what happened. Notes taken by the interviewers were later checked by an independent rater to verify that the events reported could be classified as instances of bullying operationally defined as evidence of (a) repeated harmful actions, (b) between children, and (c) where there is a power differential between the bully and the victim (Shakoor et al., 2011). Although inter-rater reliability between mothers and children was only modest (*kappa*=0.20-0.29), reports of victimization from both informants were similarly associated with children’s emotional and behavioural problems, suggesting that each informant provides a unique but meaningful perspective on bullying involvement (Shakoor et al., 2011). We thus combined mother and child reports of victimization to capture all instances of bullying victimization for primary and secondary school separately: reported as not victimized by both mother and child; reported by either mother or child as being occasionally victimized; and reported as being occasionally victimized by both informants or as frequently victimized by either mother or child or both (Bowes et al., 2013). We then combined these primary and secondary school ratings to create a bullying victimization variable for the entire childhood period (5-12 years). Children who were never bullied in primary or secondary school or occasionally bullied during one of these time periods were coded as 0 (55.5%); children who were occasionally bullied during primary and secondary school, or frequently bullied during one of these time periods were coded as 1 (35.6%); and children who were frequently bullied at both primary and secondary school were coded as 2 (8.9%).

*Physical and sexual harm by an adult.* We assessed childhood physical and sexual harm in the E-Risk Study using an approach that resembles the process undertaken by child protection agencies. Essentially this is a two-stage process. In child protection, professionals such as teachers working with children typically raise concerns if they observe signs or symptoms or if they become aware of risk that children are victims of violence. When concerns are raised, child protection officers then review the concerns and evaluate them in the context of information previously gathered on that child or family in order to determine the likelihood that abuse has taken place. In the E-Risk Study, research workers visited the home in pairs, and were extensively trained to detect signs of abuse or neglect. Each time the two research workers visited a home, they interviewed the mother using a structured interview about child harm, tested the children, and observed the family environment using the Home Observation for Measurement of the Environment (HOME; Bradley & Caldwell, 1977). If either research worker had any concerns, they flagged up the case for review. Immediately after each home visit, a review was performed if a family was flagged. In addition, at each wave, any family who had been flagged on a prior wave of the study was automatically reviewed again. The reviews were performed independently by at least 2 clinical psychologists or psychiatrists, and were based on comprehensive dossiers compiled across multiple home visits for each study member during the course of the ongoing longitudinal study.

At age 5, assessments were based on the standardised clinical protocol from the MultiSite Child Development Project Dodge et al., 1990; Lansford et al., 2002). At ages 7, 10, and 12 this interview was modified to expand its coverage of contexts for child harm. Interviews were designed to enhance mothers’ comfort with reporting valid child maltreatment information, while also meeting researchers’ responsibilities for referral under the UK Children Act. Specifically, mothers were asked whether either of their twins had been intentionally harmed (physically or sexually) by an adult or had contact with welfare agencies. If caregivers endorsed a question, research workers made extensive notes on what had happened, and indicated whether physical and/or psychological harm had occurred. Under the U.K. Children Act, our responsibility was to secure intervention if maltreatment was current and ongoing. Such intervention on behalf of E-Risk families was carried out with parental cooperation in all but one case. No families left the study following intervention.

Over the years of data collection, the study developed a cumulative profile for each child, comprising the caregiver reports, recorded debriefings with research workers who had coded any indication of maltreatment at any of the successive home visits, recorded narratives of the successive caregiver interviews, and information from clinicians whenever the Study team made a child-protection referral. Each time we visited a home, the research workers flagged concerns, and if there was sufficient evidence to code definite harm then, we did so. If evidence only met the level of probable harm, we kept an “ongoing concern list” and if, at a later wave, there was continued evidence of probable harm, or new evidence, the code was upgraded to definite harm. The profiles were reviewed at the end of the age-12 phase by at least two clinical psychologists or psychiatrists. Initial inter-rater agreement between the coders was 90% in cases for whom maltreatment was identified (100% for cases of sexual abuse), and discrepantly coded cases were resolved by consensus review. These were coded as: 0 = no physical harm at any age; 1 = probable physical harm at any age; and 2 = definite physical harm at any age. There were 15.0% of children coded as probably being exposed to physical harm and 5.1% as definitely physically harmed by 12 years of age. There were 1.5% of the children coded as being exposed to sexual abuse.

*Emotional abuse and neglect.* These forms of maltreatment were coded from research workers’ narratives of the home visits at ages 5, 7, 10, and 12. We coded quite severe examples of parental behavior observed. For example, a mother who had schizophrenia screamed and swore at the children throughout the home visit. As another example, a father who was drunk during the home visit repeatedly spoke abusively to the children in front of the research workers. We found that coders could not empirically separate emotional abuse and emotional neglect in a reliable way and thus such experiences were coded together as emotional abuse/neglect. Inter-rater agreement between the coders exceeded 85% for cases with emotional abuse and neglect, and discrepant cases were resolved by consensus review. Children with no evidence of emotional abuse/neglect were coded as 0 (88.3%), those where there was some indication of emotionally inappropriate/potentially abusive or neglectful behavior were coded as 1 (8.7%), and where there was evidence of severe emotional abuse/neglect the children were coded as 2 (3.0%).

*Physical neglect.* The cumulative observations of the physical state of the home environment documented by the research workers during home visits to the twins at ages 5, 7, 10 and 12 were reviewed by two raters for evidence of physical neglect. This was defined as any sign that the caretaker was not providing a safe, sanitary, or healthy environment for the child. This included the child not having proper clothing or food, as well as grossly unsanitary home environments. (However, this did not include a family living in a crime-ridden neighborhood for economic reasons.) Inter-rater agreement between the coders exceeded 85%, and discrepantly coded cases were resolved by consensus review. Children with no evidence of physical neglect were coded as 0 (90.9%), those for whom there was an indication of minor physical neglect were coded as 1 (7.1%), and where there was evidence of severe physical neglect the children were coded as 2 (2.0%).

**Data integrity statement**

The premise and analysis plan for this project were pre-registered on https://sites.google.com/site/dunedineriskconceptpapers/documents. Analyses reported here were checked for reproducibility by an independent data-analyst, who recreated the code by working from the manuscript and applied it to a fresh dataset.

**References**

Achenbach, T.M. (1991a). *Manual for the Child Behavior Checklist and 1991 profile*. University of Vermont.

Achenbach, T.M. (1991b). *Manual for the Teacher’s Report Form and 1991 profile.* University of Vermont.

Arseneault, L., Walsh, E., Trzesniewski, K., Newcombe, R., Caspi, A., & Moffitt, T.E. (2006). Bullying victimization uniquely contributes to adjustment problems in young children: a nationally representative cohort study. *Pediatrics, 118*, 130-138.

Bettes, B.A., & Walker, E. (1986). Symptoms associated with suicidal behavior in childhood and adolescence. *Journal of Abnormal Child Psychology,* *14*, 591-604.

Bowes, L., Maughan, B., Ball, H., Shakoor, S., Ouellet-Morin, I., Caspi, A., Moffitt, T.E., & Arseneault, L. (2013). Chronic bullying victimization across school transitions: the role of genetic and environmental influences. *Developmental Psychopathology, 25*, 333-346.

Bradley, R., & Caldwell, B. (1977). Home observation for measurement of the environment: a validation study of screening efficiency. *American Journal of Mental Deficiency,* 81, 417-420.

CACI. *ACORN user guide*. London, UK: CACI Information Services; 2006.

Danese, A., Moffitt, T.E., Arseneault, L., Bleiberg, B.A., Dinardo, P.B., Gandelman, S.B., Houts, R., Ambler, A., Fisher, H.L., Poulton, R., & Caspi, A. (2017). The origins of cognitive deficits in victimized children: implications for neuroscientists and clinicians. *American Journal of Psychiatry, 174*, 349–361.

Dhossche, D., Ferdinand, R., Van der Ende, J., Hofstra, M.B., & Verhulst, F. (2002). Diagnostic outcome of self-reported hallucinations in a community sample of adolescents. *Psychological Medicine, 32*, 619-627.

Dodge, K.A., Bates, J.E., & Pettit, G.S. (1990). Mechanisms in the cycle of violence. *Science, 250*, 1678-1683.

Downs, J.M., Cullen, A.E., Barragan, M., & Laurens, K.R. (2013). Persisting psychotic-like experiences are associated with both externalising and internalising psychopathology in a longitudinal general population child cohort. *Schizophrenia Research,* *144*, 99-104.

Fisher, H.L., Caspi, A., Moffitt, T.E., Wertz, J., Gray, R., Newbury, J., Ambler, A., Zavos, H., Danese, A., Mill, J., Odgers, C.L., Pariante, C., Wong, C.C., & Arseneault, L. (2015). Measuring adolescents’ exposure to victimization: the Environmental Risk (E-Risk) Longitudinal Twin Study. *Developmental Psychopathology,* *27*, 1399–1416.

Franklin, J.C., Ribeiro, J.D., Fox, K.R., Bentley, K.H., Kleiman, E.M., Huang, X., Musacchio, K.M., Jaroszewski, A.C., Chang, B.P., & Nock, M.K. (2017). Risk factors for suicidal thoughts and behaviors: A meta-analysis of 50 years of research. *Psychological Bulletin,* *143*, 187-232.

Horwood, J., Salvi, G., Thomas, K., Duffy, L., Gunnell, D., Hollis, C., Lewis, G., Menezes, P., Thompson, A., Wolke, D., Zammit, S., & Harrison, G. (2008). IQ and non-clinical psychotic symptoms in 12-year-olds: results from the ALSPAC birth cohort. *British Journal of Psychiatry, 193*, 185-191.

Jaffee, S.R., Caspi, A., Moffitt, T.E., & Taylor, A. (2004). Physical maltreatment victim to antisocial child: evidence of an environmentally mediated process. *Journal of Abnormal Psychology, 113*, 44-55.

Kelleher, I., Connor, D., Clarke, M.C., Devlin, N., Harley, M., & Cannon, M. (2012). Prevalence of psychotic symptoms in childhood and adolescence: a systematic review and meta-analysis of population-based studies. *Psychological Medicine, 42*, 1857-1863.

Kelleher, I., Keeley, H., Corcoran, P., Lynch, F., Fitzpatrick, C., Devlin, N., Molloy, C., Roddy, S., Clarke, M.C., Harley, M., Arseneault, L., Wasserman, C., Carli, V., Sarchiapone, M., Hoven, C., Wasserman, D., & Cannon, M. (2012). Clinicopathological significance of psychotic experiences in non-psychotic young people: Evidence from four population-based studies. *British Journal of Psychiatry, 201*, 26-32.

Lansford, J.E., Dodge, K.A., Pettit, G.S., Bates, J.E., Crozier, J., & Kaplow, J. (2002). Long-term effects of early child physical maltreatment on psychological, behavioral, and academic problems in adolescence: a 12-year prospective study. *Archives of Pediatrics and Adolescent Medicine, 156*, 824-830.

Milne, B.J., Caspi, A., Crump, R., Poulton, R., Rutter, M., Sears, M.R., & Moffitt, T.E. (2009). The validity of the family history screen for assessing family history of mental disorders. *American Journal of Medical Genetics Part B: Neuropsychiatric Genetics,* *150B*, 41–49.

Milne, B.J., Moffitt, T.E., Crump, R., Poulton, R., Rutter, M., Sears, M.R., Taylor, A., & Caspi, A. (2008). How should we construct psychiatric family history scores? A comparison of alternative approaches from the Dunedin Family Health History Study. *Psychological Medicine,* *38*, 1793-1798.

Moffitt, T.E., Caspi, A., Krueger, R.F., Magdol, L., Margol, G., Silva, P.A., & Sydney, R. (1997). Do partners agree about abuse in their relationship? A psychometric evaluation of inter-partner agreement. *Psychological Assessment,* *9*, 47-56.

Moffitt, T.E., E-Risk Study Team (2002). Teen-aged mothers in contemporary Britain. *Journal of Child Psychology and Psychiatry, 43*, 1-16.

Mortensen, P.B., Pedersen, M.G., Pedersen, C.B. (2010). Psychiatric family history and schizophrenia risk in Denmark: which mental disorders are relevant? *Psychological Medicine,* 40, 201-210.

Nock, M.K. (2009). Why do people hurt themselves? New insights into the nature and functions of self-injury. *Current Directions in Psychological Science,* *18*, 78-83.

Odgers, C.L., Caspi, A., Bates, C.J., Sampson, R.J., Moffitt, T.E. (2012). Systematic social observation of children’s neighborhoods using Google Street View: A reliable and cost-effective method. *Journal of Child Psychology and Psychiatry, 53*, 1009-1017.

Polanczyk, G., Moffitt, T., Arseneault, L., Cannon, M., Ambler, A., Keefe, R.S., Houts, R., Odgers, C.L., Caspi, A. (2010). Etiological and clinical features of childhood psychotic symptoms. *Archives of General Psychiatry, 67*, 328-338.

Poulton, R., Caspi, A., Moffitt, T.E., Cannon, M., Murray, R., Harrington, H. (2000). Children’s self-reported psychotic symptoms and adult schizophreniform disorder: a 15-year longitudinal study. *Archives of General Psychiatry, 57*, 1053-1058.

Qin, P., Agerbo, E., Mortensen, P.B. (2002). Suicide risk in relation to family history of completed suicide and psychiatric disorders: a nested case-control study based on longitudinal registers. *Lancet,* *360*, 1126-1130.

Qin, P., Agerbo, E., Mortensen, P.B. (2003). Suicide risk in relation to socioeconomic, demographic, psychiatric, and familial factors: a national register–based study of all suicides in Denmark, 1981–1997. *American Journal of Psychiatry, 160*, 765-772.

Schäfer, I., Fisher, H.L. (2011). Childhood trauma and psychosis-what is the evidence? *Dialogues in Clinical Neuroscience,* *13*, 360-365.

Schreier, A., Wolke, D., Thomas, K., Horwood, J., Hollis, C., Gunnell, D., Lewis, G., Thompson, A., Zammit, S., Duffy, L., Salvi, G., Harrison, G. (2009). Prospective study of peer victimization in childhood and psychotic symptoms in a non-clinical population at age 12 years. *Archives of General Psychiatry, 66*, 527-536.

Scott, J., Chant, D., Andrews, G., McGrath, J. (2006). Psychotic-like experiences in the general community: the correlates of CIDI psychosis screen items in an Australian sample. *Psychological Medicine, 36*, 231-238.

Seidman, L.J., Giuliano, A.J., Meyer, E.C., Addington, J., Cadenhead, K.S., Cannon, T.D., McGlashan, T.H., Perkins, D.O., Tsuang, M.T., Walker, E.F., Woods, S.W., Bearden, C.E., Christensen, B.K., Hawkins, K., Heaton, R., Keefe, R.S., Heinssen, R., Cornblatt, B.A.; North American Prodrome Longitudinal Study (NAPLS) Group (2010). Neuropsychology of the prodrome to psychosis in the NAPLS consortium: relationship to family history and conversion to psychosis. *Archives of General Psychiatry,* *67*, 578-588.

Shakoor, S., Jaffee, S.R., Andreou, P., Bowes, L., Ambler, A.P., Caspi, A., Moffitt, T.E., Arseneault, L. (2011). Mothers and children as informants of bullying victimization: results from an epidemiological cohort of children. *Journal of Abnormal Child Psychology, 39*, 379-387.

Straus, M. (1990). Measuring intrafamily conflict and violence: the Conflict Tactics (CT) scales. In: M. A. Straus & R. G. Gelles (Eds.), *Physical violence in American families: risk factors and adaptations to violence in 8,145 families* (pp.403-424). New Brunswick, NJ. Transaction Press.

Trouton, A., Spinath, F.M., Plomin, R. (2002). Twins Early Development Study (TEDS): a multivariate, longitudinal genetic investigation of language, cognition and behavior problems in childhood. *Twin Research and Human Genetics,* *5*, 444-448.

Trzesniewski, K.H., Moffitt, T.E., Caspi, A., Taylor, A., Maughan, B. (2006). Revisiting the association between reading achievement and antisocial behavior: new evidence of an environmental explanation from a twin study. *Child Development,* *77*, 72–88.

Van der Kolk, B.A., Perry, J.C., Herman, J.L. (1991). Childhood origins of self-destructive behavior. *American Journal of Psychiatry,* *148*, 1665-1671.

Varese, F., Smeets, F., Drukker, M., Lieverse, R., Lataster, T., Viechtbauer, W., Read, J., van Os, J., Bentall, R.P. (2012). Childhood adversities increase the risk of psychosis: a meta-analysis of patient-control, prospective-and cross-sectional cohort studies. *Schizophrenia Bulletin,* *38*, 661-671.

Weissman, M.M. (2000). Brief screening for family psychiatric history: the Family History Screen. *Archives of General Psychiatry, 57*, 675-682.

Werner, S., Malaspina, D., Rabinowitz, J. (2007). Socioeconomic status at birth is associated with risk of schizophrenia: population-based multilevel study. *Schizophrenia Bulletin,* *33*, 1373-1378.

Yoshizumi, T., Murase, S., Honjo, S., Kaneko, H., Murakami, T. (2004). Hallucinatory experiences in a community sample of Japanese children. *Journal of American Academy of Child and Adolescent Psychiatry,* *43*, 1030-1036.

Zammit, S., Kounali, D., Cannon, M., David, A.S., Gunnell, D., Heron, J., Jones, P.B., Lewis, S., Sullivan, S., Wolke, D., Lewis, G. (2013). Psychotic experiences and psychotic disorders at age 18 in relation to psychotic experiences at age 12 in a longitudinal population-based cohort study. *American Journal of Psychiatry, 170*, 742-750.

**Table S1.** Associations between suicidal/self-harm behaviour (SIB) and psychotic experiences (PEs) at ages 12 (T1) and 18 (T2) years with covariate paths fixed to ‘0’.

|  | **Model 1**  Baseline | **Model 2**  PEs →SIB | **Model 3**  SIB → PEs | **Model 4**  Bi-directional Free | **Model 5**  Bi-directional Constrained |
| --- | --- | --- | --- | --- | --- |
| **Model Fit** |  |  |  |  |  |
| AIC | 3553.62 | 3552.32 | 3541.11 | 3539.81 | 3537.87 |
| BIC | 3587.56 | 3591.92 | 3580.71 | 3585.07 | 3577.47 |
| ssaBIC | 3568.50 | 3569.68 | 3558.47 | 3559.65 | 3555.23 |
|  | | | | | |
| **Autoregressive paths – ORs (95% CIs)** | | | | | |
| SIB T2 on SIB T1 | 4.42  (2.53-7.75) | 4.42  (2.53-7.75) | 3.95  (2.34-6.66) | 3.95  (2.34-6.66) | 3.94  (2.35-6.61) |
| PEs T2 on PEs T1 | 5.95  (3.05-11.61) | 5.46  (2.82-10.61) | 5.96  (3.06-11.62) | 5.47  (2.82-10.61) | 5.53  (2.87-10.65) |
|  | | | | | |
| **Cross-lagged paths – ORs (95% CIs)** | | | | | |
| SIB T2 on PEs T1 | --- | --- | 2.46  (1.56-3.87) | 2.46  (1.56-3.87) | 2.51  (1.64-3.83) |
| PEs T2 on SIB T1 | --- | 2.82  (1.15-6.92) | --- | 2.82  (1.15-6.92) | 2.51  (1.64-3.83) |

AIC = Akaike Information Criterion; BIC = Bayesian Information Criterion; CIs = confidence intervals; ORs = odds ratios; ssaBIC = Sample-Size Adjusted BIC. All models statistically adjusted for the non-independence of twin observations and covariates = sex; childhood victimisation; family history of suicide; family history of psychiatric disorders; externalising problems; internalising problems; family socio-economic status.
